# User Centered Virtual Coaching for Older Adults at Home Using SMART Goal Plans and I-Change Model

**DOI:** 10.3390/ijerph18136868

**Published:** 2021-06-26

**Authors:** Andoni Beristain Iraola, Roberto Álvarez Sánchez, Santiago Hors-Fraile, Despoina Petsani, Michail Timoleon, Unai Díaz-Orueta, Joanne Carroll, Louise Hopper, Gorka Epelde, Jon Kerexeta, Panagiotis D. Bamidis, Evdokimos I. Konstantinidis

**Affiliations:** 1 Digital Health & Biomedical Technologies Department, Vicomtech Foundation, Basque Research and Technology Alliance (BRTA), Mikeletegi 57, 20009 San Sebastián, Spain; ralvarez@vicomtech.org (R.Á.S.); gepelde@vicomtech.org (G.E.); jkerexeta@vicomtech.org (J.K.); 2 e-Health Department, Biodonostia Health Research Institute, Paseo Dr Begiristain s/n, 20014 San Sebastián, Spain; 3 Salumedia Labs, Research Division of Adhera Health, Inc., Palo Alto, CA 94304, USA; shors@adherahealth.com; 4 Lab of Medical Physics and Digital Innovation, School of Medicine, Aristotle University of Thessaloniki, 54124 Thessaloniki, Greece; despoinapets@gmail.com (D.P.); mtimoleon@auth.gr (M.T.); pdbamidis@gmail.com (P.D.B.); evdokimosk@gmail.com (E.I.K.); 5 Department of Psychology, Maynooth University, Maynooth W23 F2H6, Co. Kildare R51, Ireland; unai.diazorueta@mu.ie; 6 School of Psychology, Dublin City University, Dublin 9, Ireland; joanne.carroll@dcu.ie (J.C.); louise.hopper@dcu.ie (L.H.); 7 WITA SRL, 38123 Trento, Italy

**Keywords:** virtual coaching, data visualization, incremental machine learning, feature engineering, self-management, healthy and active aging

## Abstract

Preventive care and telemedicine are expected to play an important role in reducing the impact of an increasingly aging global population while increasing the number of healthy years. Virtual coaching is a promising research area to support this process. This paper presents a user-centered virtual coach for older adults at home to promote active and healthy aging and independent living. It supports behavior change processes for improving on cognitive, physical, social interaction and nutrition areas using specific, measurable, achievable, relevant, and time-limited (SMART) goal plans, following the I-Change behavioral change model. Older adults select and personalize which goal plans to join from a catalog designed by domain experts. Intervention delivery adapts to user preferences and minimizes intrusiveness in the user’s daily living using a combination of a deterministic algorithm and incremental machine learning model. The home becomes an augmented reality environment, using a combination of projectors, cameras, microphones and support sensors, where common objects are used for projection and sensed. Older adults interact with this virtual coach in their home in a natural way using speech and body gestures on projected user interfaces with common objects at home. This paper presents the concept from the older adult and the caregiver perspectives. Then, it focuses on the older adult view, describing the tools and processes available to foster a positive behavior change process, including a discussion about the limitations of the current implementation.

## 1. Introduction

According to data from World Population Prospects: the 2019 Revision presented by the United Nations [1], by 2050, one in six people in the world will be over age 65 (16%), up from one in 11 in 2019 (9%). By 2050, one in four persons living in Europe and Northern America could be aged 65 or over. In 2018, for the first time in history, persons aged 65 or above outnumbered children under five years of age globally. The number of persons aged 80 years or over is projected to triple, from 143 million in 2019 to 426 million in 2050. Despite living longer, natural age-related decline combined with the frequent comorbidity [2] may require that the older adults have some degree of support and supervision. Furthermore, most older adults prefer living on their own instead of staying in a healthcare institution, which does not guarantee better outcomes than at-home care [3]. This has fostered policies to promote active and healthy aging, trying to delay or even avoid dependency, and being able to enjoy this stage in life as a healthy individual.

Coaching is a form of counseling to maximize personal potential, according to the ECVision funded by the European Commission [4], and the coaching strategy includes a plan of actions or guidelines. Optimal guidance is achievable when the coach is aware and adaptable to the coached person’s context, goals and preferences. Preventive and online care must deal with long-term user monitoring and support, maintaining user adherence. The role of a coach is very important in this sense to motivate the user and set, track and adapt training plans with specific goals in mind. This coach needs to have access to detailed data about the user status, interests, behavior and goals to properly guide the user. Unfortunately, in practice it is not feasible to have this kind of support for most people due to limitations in the healthcare services.

As a solution to this limitation, virtual coaching tries to attain similar support to that of a human coach. Siewiorek [5] defined a virtual coach system (VCS) as an always-attentive personalized system that continuously monitors the user’s activity and surroundings, and delivers interventions—e.g., intentional messages—when these are desired. Coaching interventions for older people to adhere to healthier habits could prolong their independence and relieve costs from healthcare systems.

Traditional coaching has been seen as a human-to-human relationship. Yet, this concept is shifting as technology becomes part of our lives. The literature review conducted by Wolever et al., 2013 [6] found that only 5% percent of the analyzed studies about health coaching were completely technology based. Further, those traditional technology-based behavior change systems were implemented as simple if-then-else algorithms like decision trees, with limited potential for adaptation and self-learning from participants’ feedback. Early works such as Bacciu et al. (2015) [7] focus on user interface and Internet of Things aspects for a VCS at home. This is also the case for the study by Gordienko et al. (2017) [8], which also focused on the same aspects, but they are far from Siewiorek’s vision about what a VCS constitutes. In a recent survey, Lete et al. (2020) [9] also found limited technological development on virtual coaching for older adults. More recently, thanks to several research projects funded in 2017 by the European Research and Innovation Action (RIA) on virtual coaching for older adults, more technologically sophisticated systems have started to emerge. This is the case of the EMPATHIC project [10], focused on natural language speech interaction enhanced with emotional cues, where the coach continuously adapts to previous interactions and provides support on several aspects of older adults’ well-being. Council of coaches [11] also focused on natural language interaction but included the concept of embodied conversational social characters. Supporting Active Ageing through Multimodal coaching (SAAM) [12] stresses the participation of the older adult social circles in the coaching process and social activity to combat isolation. Virtual Coaching Activities for Rehabilitation in Elderly (vCare) [13] considers tele-rehabilitation and frailty aspects supported by gamification and advanced vital sign monitoring. The Novel Empowering Solutions and Technologies for Older people to Retain Everyday life activities (NESTORE) [14] project certainly underlines the importance of behavior change models and carries a systematic review of virtual coaching for well-being (2020). This review tried to analyze these areas: (i) the different coach definitions, (ii) the behavior change models used, (iii) which domains are tackled and (iv) the technical implementation of the system. It concludes saying that there is a trend for the convergence about what a virtual coach for older adult well-being should be and which domains it should tackle.

Coaching implies fostering behavior change processes in the person, therefore it is important to consider a behavior change model to guide this process. The Integrated Change Model (I-Change Model) is a behavior change model derived from the attitude–social influence–self-efficacy model [15] which states that covert and overt behaviors are determined by a person’s motivation or intention to carry out a particular type of action, originated by awareness factors (e.g., knowledge, risk perceptions and cues to action). This sequence from awareness to motivation, action and behavior depends on the person’s preceding factors (e.g., biological, psychological, previous behavior, social and environmental) and information factors (e.g., message, channel, source). The I-Change model has been successfully blended with technology to promote healthy behaviors in several domains, such as smoking cessation [16], hypertension reduction [17] and healthy lifestyle in general [18], among others. Therefore, it seems well suited for the purpose of promoting these behaviors under the umbrella of a VCS. This is in line with a pragmatic methodology to design digital health programs which was recently published, in which the interventions are strongly guided by goals and objectives [19].

According to Turner-Stokes [20], every individual sets lifelong objectives that are considered goals that take a long time to achieve (i.e., years). These long-term goals can be split into smaller medium-term achievements or intermediate milestones and short-term objectives, which permit measuring self-evolution. Finally, those short-term objectives can be translated into a plan with individual action items. Following this idea, short-term objectives become specific and the progress can be measured. Goals must be achievable for the person, action-oriented, realistic, relevant, and finally, time-limited. These features inspire the acronym of a SMART (specific, measurable, achievable, relevant and time-limited) goal. For example, a SMART goal might be [21]: “Being able to walk a quarter of mile to reach my local supermarket within six weeks.” Tolchin et al. [22] proved that SMART goals for treatment planning in psychotherapy were more beneficial than other alternatives.

**Figure 1:**
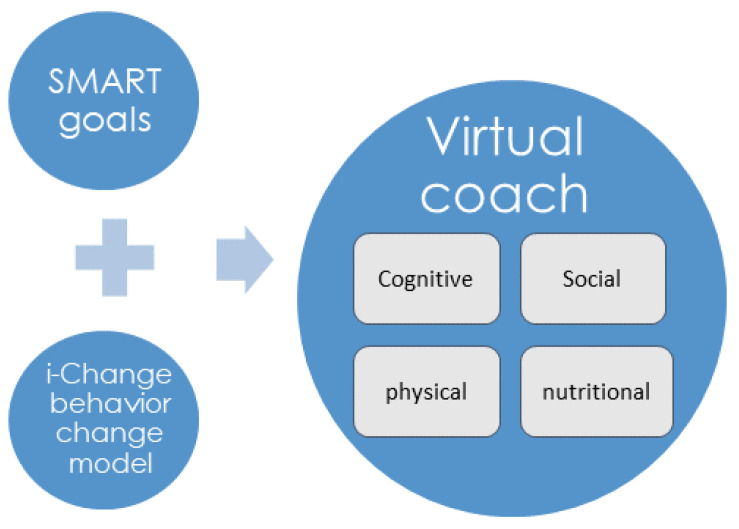
I-Change and SMART coaching.

In this context, H2020 CAPTAIN is a research and innovation action project which pursues the creation of a virtual coach system to support older adults living independently at home [23,24]. This VCS proposes a digitalization of the I-Change model that will guide older adults towards the successful achievement of SMART goals as part of their lifelong objectives (Figure 1), circumscribing them into four main dimensions: cognitive, social, physical and nutritional.

This paper presents the coaching aspects in CAPTAIN from different perspectives following a top-down approach. Section 2 describes the overall virtual coaching concept and ecosystem proposed and gives an overview of the features provided from the older adult perspective and from the caregiver perspective. Then, in Section 3 the whole coaching process is described from the older adult side, presenting the available tools and processes to foster a positive behavior change process. Section 4 presents a detailed review of the coaching intervention tools. Section 5 evaluates the VCS, and ultimately Section 6 summarizes the final conclusions.

Some of the preliminary ideas and user interface were presented at the 2020 International Conference on Multimodal Interaction (ICMI 2020), in the Multimodal e-Coaches workshop [24]. The current work presents a refined version and describes in detail several technical aspects not presented before. The main contributions of this paper over earlier works are:

- An updated review of state-of-the-art virtual coaching for older adults’ well-being.
- An in-depth and consolidated description of the complete CAPTAIN ecosystem, also considering the caregiver perspective.
- A detailed description about how the I-Change and SMART goal concept have been integrated and implemented as the cornerstone pieces of the VCS.
- A precise specification about how the day plan is built in three stages: weekly, daily and momentary.
- An enhanced incremental machine learning-supported algorithm for momentary intervention planning.
- The addition of the analytics aspects of the coach, highlighting its relevance to (1) properly estimate motivational insights and (2) create goal performance statistics to enhance the adherence to the system.
- A discussion on the limitations and future work.

## 2. CAPTAIN Virtual Coaching Ecosystem

### 2.1. Overall Coaching Approach

The CAPTAIN virtual coaching ecosystem is focused on supporting behavior change processes of older adult people towards active and healthy aging (AHA), to extend independent living not only quantitively but also qualitatively. Effective coaching is a complex process that additionally requires close cooperation between at least healthcare and social services, as well as the community, family and friends, including formal and informal caregivers. Furthermore, it must foster a user’s empowerment by placing them at the center of the decision-making process.

**Figure 2:**
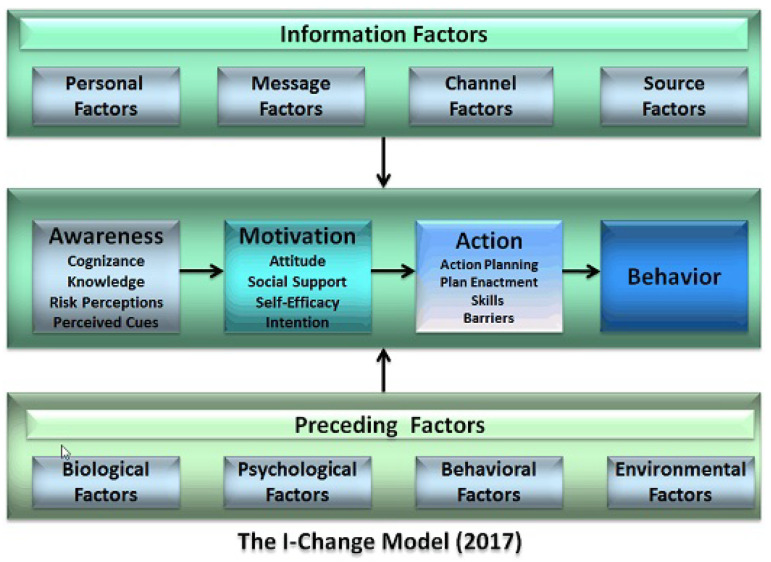
I-Change behavior change model [25].

Our proposal to properly support this complex scenario is founded by digitally implementing two main concepts:

(1) The I-Change model, previously introduced in Section 1, as the reference framework to achieve optimal behavior change. This contrasted model will act as a main conductor which guides our system workflow at every moment. More specifically, we have mainly focused on providing users with tools that will guide them towards the successfully consecution of the three pillar phases described by the I-Change model: awareness, motivation and action (Figure 2).
(2) As a vehicular instrument that guides the process, we based the digitalization of care plans on SMART goals, as previously described in Section 1. This concept represents a proper choice since its foundations correlate with the selected methodology:
  - Specific: the specific nature of SMART goals helps the system to better design the action planning suggested by I-Change.
  - Measurable: this feature allows to achieve the awareness and motivation phase while evaluating the impact of the actions and behavior change.
  - Achievable: this characteristic also is in line with the motivation phase, but correlates with the selection of proper actions and their intensity.
  - Relevant: the awareness phase relies on highlighting the relevancy of the care plans for the older adult, who is in the center of the coaching process.
  - Time-limited: this feature is also key for the action planning as well as for the motivation phase.

As presented earlier, the virtual coaching ecosystem (VCE) puts the older adult in the center of the coaching process. Older adults might be living independently in their home or supported by caregivers. Each older adult decides which SMART goals to have and their personalization, either on their own or after a discussion with their caregivers.

Finally, the VCE also allows caregivers to work not only individually with users, but also at the population level by designing SMART goal plans tailored to the population in a dynamic way. Caregivers can see the evolution of the population characteristics and group them, then evaluate the impact of the designed plans and improve them. Under this paradigm, the virtual coach system can also suggest SMART goals to older adults based on the effectiveness on other older adults with similar characteristics. Thanks to this approach, population grouping, plan design and validation, as well as coaching plan delivery, can be improved iteratively based on the collected feedback.

### 2.2. Physical Platform at Home

As presented in the introduction, the virtual coach needs to be able to track the user and the environment and to interact with them in the most natural manner to deliver the interventions. In this section we present how these aspects were implemented in the H2020 CAPTAIN project by describing the overall architecture under which the virtual coach was implemented.

**Figure 3:**
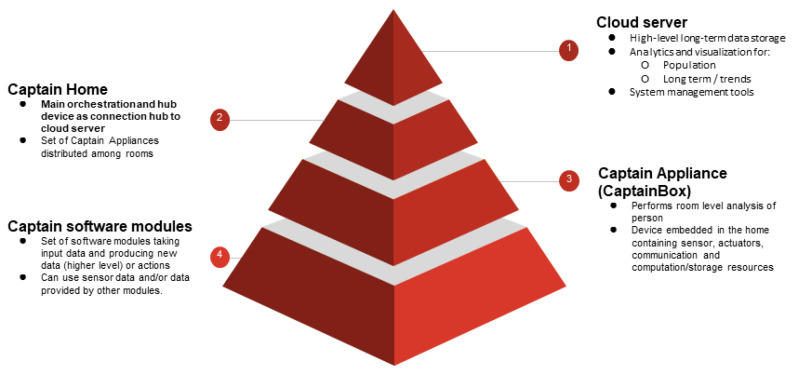
Physical view of CAPTAIN coaching platform.

The CAPTAIN platform is composed of local hardware and software deployment at each older adult’s home and a central server-side platform (Figure 3). The local and server-side parts of the platform interact using a REST API [26].

The server side provides long-term and high-level aggregated and anonymized data storage, analytics and visualization on the stored data and system management tools, including software updates.

The local side at each home follows a distributed and extendable design approach. Nevertheless, there is always a master device node, taking care of home orchestration and taking the role of hub with the server side. This device is called the CaptainBox, and it is implemented using a Raspberry Pi 4 device with 4GB memory and a 64GB SD card. It also includes a stereo microphone and speakers, as well as a camera and pico-projector. All these devices are integrated in a 3D-printed case so that the device is self-contained. The rest of the devices at home contain a subset of these features and extend the sensing and interface range to all rooms at home. There is an additional hardware which is the MentorAge^®^ device, produced by the Nively company (partner in the CAPTAIN project), which provides 3D detection of the user location as well as the detection of user actions, protecting user privacy. Depending on the hardware components that each additional device contains, devices are categorized as either CaptainSatelliteCamera or CaptainSatelliteProjector. The first contains a MentorAge^®^ while the second contains a pico-projector, but both include stereo microphones and speakers and limited computing capabilities.

Regarding the software components, they are distributed among the devices and interact with them using a publish-subscribe messaging paradigm.

The older adult interacts with the system using speech and video projections in different locations at each room at home, with multi-language support. This user interface is supported by the following components:

- Automatic speech recognition (ASR) of several commands for interacting with the system: numbers, yes/no and certain key words.
- Speech synthesis for interacting with the user (text to speech module, TTS).
- Narrow field of view with 3D-tangible video projection where the user can touch the surface where the GUI is shown to interact with the controls (e.g., buttons).
- 2D video projection on objects and walls at home, supported by body gesture recognition (linked to the sensing capabilities).

The CAPTAIN platform provides the following sensing capabilities:

- User location in 3D space at each room, including gait features, posture recognition and identification of dangerous postures
- User-tangible interaction with physical objects in the room
- Face detection
- Facial recognition and authentication at home
- Facial expression recognition for emotion estimation
- Speech analysis for emotion recognition
- Environment weather sensing

The VCE continuously monitors the user and environment status to provide an aggregated and updated view of any user recognized by the system as the coached person, including its location at home and in the room, the object they are interacting with, the speech command they said and their facial expression. In addition, the environment status provides information such as the weather in the home surroundings. All this sensing permits the preceding factors and information factors in the I-Change model, gaining knowledge about the user and permitting more personalized interactions.

The coach uses the app dispatcher component to identify the most suitable device to provide the user interface to the user based on his location at home and requests it to launch an intervention tool (e.g., Agenda), through the instance of the tool projected GUI component running in that device. This component manages the interaction with the user and the business logic of the intervention tool while providing a unified graphical user interface (GUI) for any intervention tool. After finishing the intervention, the tool-projected GUI reports the to coach that the intervention is finished, along with its outcomes.

### 2.3. Older Adult Perspective

The overall idea of this virtual coaching system from the participant (i.e., older adult) perspective is to become a lifelong learning (i.e., coaching), motivational and supportive platform for them with the purpose of fostering healthy habits through a behavior change process executed themselves.

Long-term engagement and adherence to the plans are two of the key issues within this kind of approach that are completely related to the motivation phase suggested by the I-Change model. The way in which our system deals with these challenges is threefold: first, by splitting broad, long-term objectives into time-bound, specific milestones that we call SMART goals. Second, each of the SMART goal plans dynamically adapts to the user’s availability and capabilities (given certain plan constrains to guarantee a behavior change). Finally, by providing the user with periodic feedback about his progress and presenting motivational messages highlighting his achievements so that the user can have a sense of succeeding and can keep track of the evolution (i.e., accomplish the motivation phase).

**Figure 4:**
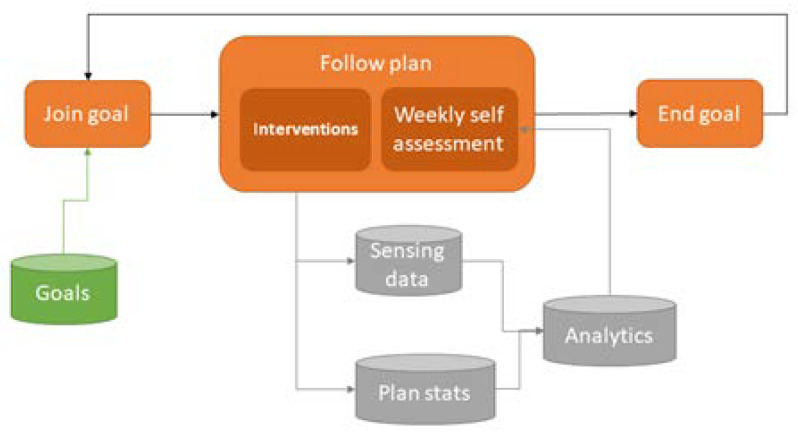
Older adult perspective of the virtual coaching ecosystem.

The coaching process itself is presented in detail in Section 3: virtual coach system. Nevertheless, it is important to highlight some aspects of this approach. Users can join any SMART goal from the pre-populated catalog (only if the older adult fulfills all its requirements). Every SMART goal contains a predefined initial intervention plan scheduled by weeks. The system stores and analyzes sensing data to choose the best moment to deliver the intervention plan. All along this process, the system, in addition to the sensing data, also stores goal-related performance and engagement. Using statistical and AI tools, the coach can evaluate the user status and surroundings to compare it with his achievement in the goals to consequently adapt the coaching process. Goal plans can also evolve as described in next section, based on population-level analytics comparing the user status before and after performing the goal plans. This process is illustrated in Figure 4.

### 2.4. Caregiver Perspective

From the caregiver perspective, the VCE provides support in the process of providing care in a more personalized, effective and efficient way iteratively. Different types of caregivers and institutions can take part in this process.

**Figure 5:**
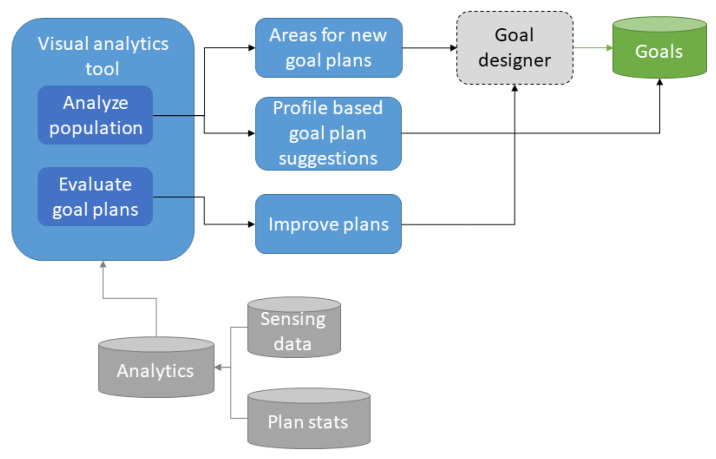
Caregiver perspective of the virtual coaching ecosystem.

The main concept is that thanks to the VCE, caregivers can analyze the participant (i.e., older adult) population in a structured manner by grouping similar individuals and analyzing their evolution to designed SMART goal plans. Then, they can evaluate the effectiveness of the plans they design. This process becomes a loop in which existing SMART goal plans are refined and replaced by better plans and new plans are generated as the population evolves, new users are added and new coaching needs are identified. In addition, certain goals can be suggested to the participants based on their effectiveness on other similar participants with similar interests. These ideas are presented in Figure 5. The technical details of this stratification tool are out of the scope of this paper.

## 3. Virtual Coach System (VCS)

This section describes the virtual coach system from the conceptual to the technical aspects. The visual analytics clustering and plan definition tool is out of the scope of this paper.

The VCS focuses only on SMART goals and the I-Change behavior change model, as described later in this section.

Every SMART goal is composed of a weekly plan with coaching actions provided by digital intervention tools such as recommender systems, quizzes, trainers and games. All of them are focused on supporting a behavior change process. The scope of coaching covers these specific areas or dimensions: cognitive, physical, social and nutritional.

One key aspect that the VCS focuses on is to simplify the self-empowerment process. In CAPTAIN, the participant (i.e., older adult) has an active role in two levels. First, it is he (on his own or supported by a caregiver) who decides which SMART goal to join from a catalogue (created by experts in the area and described later in this section), including the selection of the most suitable level of difficulty for his needs. Then, whenever the coach suggests an intervention to the participant, he can postpone it and the coach will try to adapt the plan to meet the SMART goal planning.

Participants interact with this virtual coach at home using a combination of 2D- and 3D-tangible projections, supported by natural language processing. This system is also able to sense the participant’s location and body motion, recognize his facial expression and even identify him thanks to the combination of several image and audio analysis techniques. The devices forming the CAPTAIN system are blended into the participant’s home, mimicking conventional furniture and appliances.

### 3.1. I-Change Model Application

As introduced in Section 2.1, the I-Change model has inspired several aspects of our VCS, focusing on digital support as its cornerstone: awareness, motivation and action phases (Figure 2). To tackle awareness, each SMART goal plan includes tips to learn about the aspects in which the goal focuses, such as physical activity. Then, there are several intervention tools (presented in Section 4) which lie among one of the three phases: awareness, motivation and action. The coffee session intervention tool promotes periodic self-reflection, where the user can check his progress throughout the SMART goal plans and receive suggestions for next goals. The motivational day agenda shows motivational messages when the user achieves certain milestones when completing the target day plan. The motivational day insights provide daily motivational messages when the user has either a personal or SMART goal-related positive evolution. Most of the intervention tools try to provide positive feedback of completion when succeeding. Finally, the main purpose of the coach is to trigger actions, which are interventions contained in the SMART coaching plans in the most suitable circumstances.

Information factors, also depicted in Figure 2, are covered by using natural user interfaces based on speech technologies and tangible projections on top of common objects at home. Simple graphical user interfaces, appropriate speech volume and content density, provides the possibility of personalizing many setup parameters in the system.

Regarding preceding factors, they are considered requirements for joining each SMART goal by describing the effort required and the minimum health requirements to meet the plan, as well as by adapting certain coaching interventions to the environment of the user. For example, considering the weather in the neighborhood for suggesting outdoor activities.

### 3.2. SMART Goals and SMART Goal Plugins

SMART goal plugins are care plans aimed to reach a very specific goal regarding any of the VCS coaching dimensions (physical, cognitive, social or nutritional) in a specific period (i.e., range of 4–8 weeks), following the SMART goal concepts. They must rely on digital intervention tools which are pieces of software that will be detailed in Section 4. A SMART goal plugin is a self-contained set of data including weekly intervention plans, trigger rules for the interventions, and configuration parameters so that the participant can choose the difficulty of the goal. The idea is that third-party institutions and companies can create their own specific SMART goal plugins and make them available for the participants to join. This opens the possibility of a market niche in which participants could offer their own of plans to other participants in exchange for microtransactions.

The coach relies completely on these plugins to schedule and dispatch interventions to the participant. Consequently, the responsibility derived from the amount and intensity of the coaching interventions provided to a given participant by the coach following a SMART goal plugin falls to both the author of the SMART goal plugin as well as in the participant who accepted to follow the recommendations enumerated within the plugin, instead of the VCS itself.

When a participant selects and configures a SMART goal plugin, a SMART goal plugin instance is created with the specific parameters for that goal selected by the participant.

From the participant perspective, he has access to a broad catalogue of SMART goals, describing their focus (e.g., increase fiber intake habits in eight weeks), requirements (e.g., not having Crohn’s disease) and an overview of the expected effort from the participant to fulfil it. The participant can join a goal in a certain difficulty level (which affects the length and activities in the plan) and once he finished the plan, join another or even the same (the coach suggests a suitable new difficulty for the same goal based on his performance). With this approach, we combine short-term commitment and follow up to very specific goals with long-term progression.

**Figure 6:**
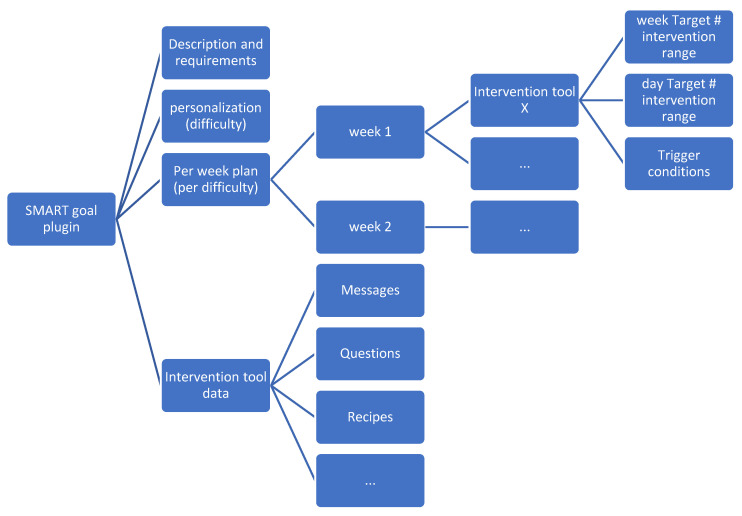
SMART goal plugin structure schema.

Each SMART goal plugin plan follows the structure shown in Figure 6. It contains:

- A description of the goal and the requirements to join it.
- The difficulty personalization.
- The weekly plans for each of the difficulty levels including the type and minimum and maximum number of interventions to carry out, as well as some trigger conditions.
- All data required by the intervention tools in the weekly plan to produce the proper interventions.

### 3.3. Dynamic Coach Planning of SMART Goal Plans

**Figure 7:**
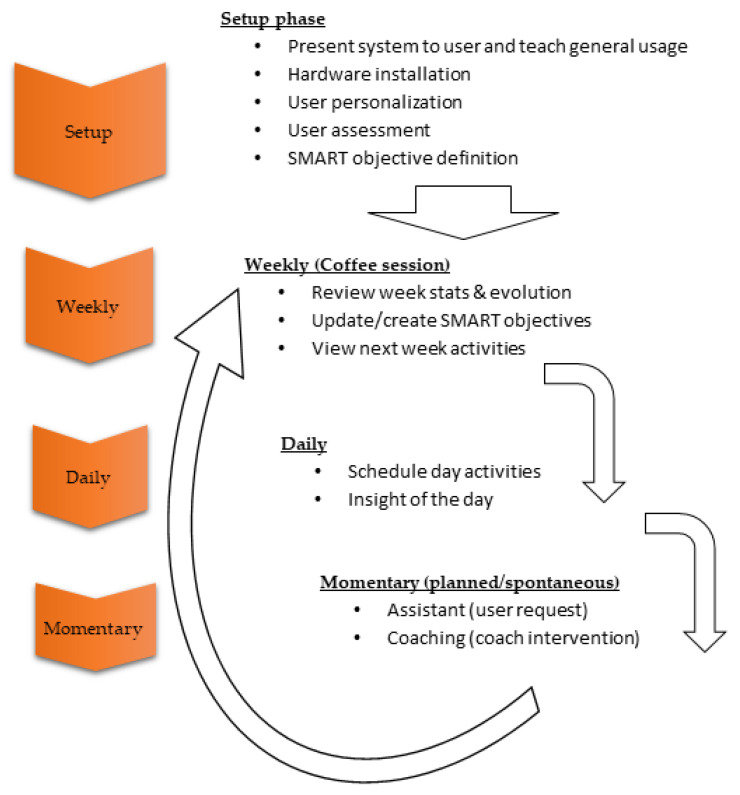
Proposed older adult coaching framework.

The coaching framework proposal is depicted in Figure 7. The system is installed in the participant’s home where the hardware is tuned, the system personalized, and the participant is assessed regarding the different coaching dimensions. This assessment is useful for the clustering process and to get a baseline to measure the evolution. Once the system is installed, the coach automatically launches a coffee session for the participant to join one or several SMART goals. After the participant has joined any SMART goal, the coach starts delivering interventions based on those goals.

The VCS works in three timescales—week, day and momentary—using intervention tools linked to each of these timescales (Figure 7)**.**

Once a week, the coach suggests the participant to have a coffee session. The participant can decide to postpone this intervention, just like any other intervention. This is an intervention tool for the participant to reflect on the past week and commit to the next week’s plan. First, the participant can see several simple charts showing his performance regarding the rate of activities carried out from the active goal plans as well as the overall scores. This is meant to be a motivational step. After this step, the participant can join additional goals or drop active goals if the participant decides to do so. Finally, the participant can see a summary of the activities planned for the next week. The coffee can also be requested by the participant, and it is automatically launched if the participant has no active goals.

Every day when the participant is detected at home, the coach shows the agenda for the day containing the set of intervention tools for that day as well as the minimum and maximum amount with the purpose of achieving the SMART goal plugin demands. Then, the coach shows daily insights if they exist. These interventions can be of three kinds: (a) a piece of information to increase the awareness about any active goal, (b) a motivational message about the participant’s performance yesterday compared to historical data, (c) a motivational message regarding his mood or activity at home, not linked to any goal.

After the agenda and insights have been delivered to the participant, the rest of interventions can take place. Since the coach does not have access to the participant’s calendar, it does not know in advance when the participant will be available at home. Therefore, whenever the participant is detected by the system, it tries to balance between delivering the maximum number of interventions to maximize daily activities while not being overwhelming for the participant. To do that, the coach performs a dynamic ranking of the pending activities based on several aspects and waits between interventions based on the priority value of the highest ranked intervention combined with an incremental learning (i.e., type of machine learning) algorithm personalized for the participant, which can decide to skip delivering an intervention in a certain moment since it anticipates that the participant would reject it.

When the coach decides to deliver an intervention, it relies on external intervention tools, which are described in Section 4.

#### 3.3.1. Weekly Plan

Coaching is completely based on the SMART goal plugin plans already presented in this section which contain per week target activities (i.e., milestones). The coach is responsible for supporting the participant in achieving those weekly milestones by creating adaptive per day activity plans and distributing them appropriately during the day using a momentary planning algorithm.

By combining the static per week plans with the dynamic per day and momentary planning, we can have the best of both approaches. On the one hand, we can guarantee that the participant receives a validated coaching plan including the fixed maximum and minimum intensity ranges so that they are safe and effective for the participant. On the other hand, the coach can dynamically distribute the interventions through the week and day to adapt the dispatching to his habits and balance the target of achieving the maximum intensity with not being too overwhelming for the participant.

#### 3.3.2. Day Plan Generation

Every day the coach reviews all the participant’s active goals to create a combined day plan. The day plan consists of a list of intervention tools to be launched during the day, as well as the minimum and maximum number of times to be launched. It can also include certain requirements to launch, such as specific time ranges or trigger conditions (e.g., sitting on the sofa for more than 30 min). The adaptive algorithm that builds this daily plan for every user is described in Section 3.4.

#### 3.3.3. Momentary Planning

Once the day plan has been generated, the coach will try to deliver all the interventions in the plan to the participant throughout the day. This momentary planning must also strike a balance between achieving the maximum number of interventions in the agenda and keeping the participant engaged without being overwhelming. In addition, the coach cannot know in advance when the participant will be available at home since it cannot have access to the participant’s calendar.

Therefore, the coach has been designed to solve the next problem description.

Goals:

- Primary: To achieve the minimum target intervention set for the day.
- Secondary: To achieve the maximum target intervention set for the day.

User-friendly restrictions:

- Avoid launching all interventions in a row.
- Avoid launching the same type of intervention tool twice in a row.
- Adapt to the participant’s behavior.

Intervention tool restrictions:

- Time range-based restrictions.
- Trigger condition required to launch an intervention.
- Intervention tool priorities.

Limitations for the momentary coach:

- Cannot know in advance when the participant will be at home.
- Cannot know the participant’s preference for doing certain interventions in certain situations.
- If a participant rejects a certain intervention, the coach does not know why. It could be because it is not the proper situation, moment of the day or intervention tool. Therefore, it does not know if it should insist or not and when.

Based on the problem description, the proposed algorithm performs as follows. Each time the participant is detected by the sensing system or his status changes over time, the coach evaluates if there is any intervention that could be launched and decides whether it is a good moment to launch it or not. If the participant is already doing another intervention, the coach sticks to the previous intervention unless the participant leaves the room. In that case, the intervention is considered rejected by the participant, so it can be launched again later. The algorithm follows two steps: (a) selecting the most suitable intervention from those pending in the day plan and (b) deciding if the moment is appropriate to launch it.

To select the most suitable intervention, only those interventions which can be launched under current circumstances are ranked. This filtering removes those intervention tools which have already achieved the maximum number or those which cannot be triggered, either because it is not the proper time range (according to the plan) or it contains certain trigger conditions that are not met in this moment (e.g., person touching the fridge). The priority ranking formula uses a linear combination of several parameters, as presented:

$$\begin{array}{cc} priority\;ranking & \\ & =1.1*participant\;requested+0.6\left(not\;minimum\;target\;interventions\;reached\right)\\ & +0.2*\left(it\;was\;not\;the\;previous\;intervention\right)+0.1\\ & *\left(intervention\;requires\;condition\right)+0.06*\left(1-\left(remaining\;time/\left(time\;range\right)\right)\right)\\ & +0.04*\left(1-accomplishment\;rate\right) \end{array}$$

$$acomplishment\;rate=\{\begin{array}{c} 1\;if\;(carried\;out\;interventions>=minimum\;target\;interventions)\;\\ \frac{carried\;out\;interventions}{minimum\;target\;interventions},\;otherwise \end{array}$$

where *participant requested* is a Boolean value which is one if this intervention has been requested by speech command and zero if not. The rest of the Boolean parameters are coded with the same representation.

This formula gives top priority to interventions directly requested by the participant (*participant requested*). Then, it gives more priority to intervention tools which have not reached the minimum target (*not* *minimum target interventions reach*). It tries to avoid launching the same intervention tool twice in a row (if *it was not the previous intervention*) and gives more priority to those which require a trigger condition (*intervention requires condition*). Finally, it gives more priority to those intervention tools which have less of a time range available to be launched. The set of parameters and their importance (i.e., weight in the linear combination) were obtained empirically after discussion with non-technical partners in the CAPTAIN project.

To decide if this is an adequate moment to deliver an intervention to the participant, two tests are carried out. The first one is checking if “enough time” has passed from the last intervention to avoid overwhelming the participant. This inter-intervention time is based on the priority of the selected intervention. Therefore, in the component configuration, we can set different waiting times for different intervention priority value ranges. Thanks to this mechanism, the coach can be more insistent for interventions with higher priority and less for less important ones. For the second check, we use an incremental machine learning model which tries to figure out if the participant will accept a certain intervention in the status before actually delivering it. In any case, since this machine learning algorithm could end up biased by the participant’s behavior, for example, by rejecting all interventions, it cannot cancel more than a configurable number of interventions in a row.

**Figure 8:**
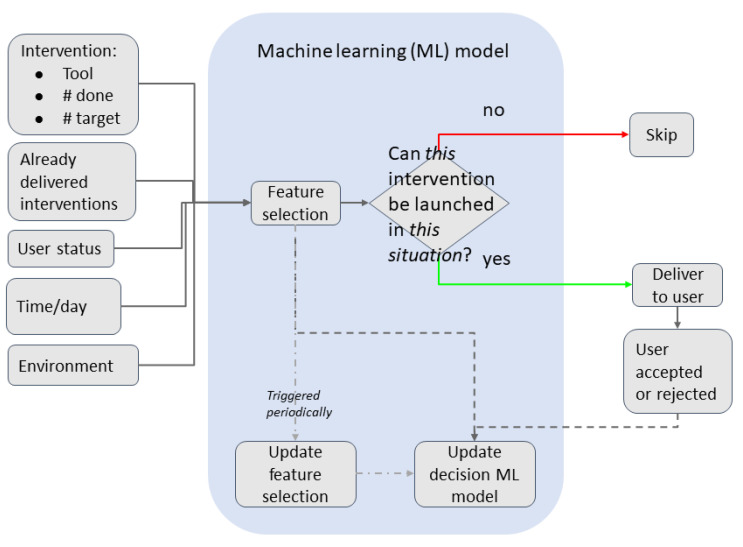
Incremental learning ML model training and inference.

The incremental machine learning model learns each participant’s patterns for accepting and rejecting interventions. The input is the participant status when the intervention is about to be delivered, timing information, historic information about previous rejected interventions and environment information, as shown in Figure 8. With these features, the model predicts if it is a good moment to launch that intervention or not with certain probability. There is an intermediate feature selection step to improve the accuracy of the model, which is also dynamically adapted to each specific participant.

One of the characteristics of this model is that it uses incremental learning, which means that it is continuously improving and adapting as the participant carries out new interventions or rejects them.

Finally, the feature selection itself adapts to the participant behavior. Feature selection is a common technique in machine learning to improve the model’s accuracy for simple models and a low amount of data points. Each time a participant finishes an intervention, all features described in this section are stored in a local database, including if the participant accepted or rejected that intervention. Periodically, when enough new samples are available, the feature selection algorithm is trained to use the most meaningful features from all collected for that participant.

### 3.4. Coach Analytics

The virtual coach performs these batch computation data analysis processes periodically:

- Day plan generation: this module generates the day plan introduced in Section 3.3.2 daily. To do so, it combines information from three different sources: (1) the corresponding goals a user is subscribed to, (2) the weekly configuration of those goals and (3) the set of interventions the user has already carried out. The planification process can be described as follows: between the first half of the week, the coach evenly distributes the weekly interventions to try to achieve the maximum number of interventions set by the goal configuration. From Thursday onwards, if the person has been able to easily reach the minimum set of interventions, the algorithm keeps pushing the participant towards the maximum but if the participant has not been able to keep up with the plan, the target is lowered towards the minimum. This algorithm has been designed to balance between achieving the maximum and keeping the user motivated.
- Goal performance statistics and chart generation: to keep the user updated as well as motivated as much as possible, goal performance statistics are frequently generated. These statistics are translated into charts that can reflect progress in two different levels: (1) high-level charts that depict the performance related to the whole goal duration (long memory) and (2) low-level charts that show the most recent performance (short memory). Both types of charts unveil information in a quantitative (how many interventions have they completed compared to the schedule?) as well as qualitative (how well do they complete them?) manner. All these charts are presented trying to boost the user adherence and motivation, so they attempt to always deliver the information in the kindest/most optimistic way possible. They automatically appear during the coffee sessions explained in Section 4.1.1.
- Motivational message generation: from the daily usage of the system, a lot of information related to the user’s general status as well as to the interventions they periodically perform as part of their goals is collected. These two sources of information are analyzed to generate insights that could provide some insight or even used as a source of motivation for the user. These insights are translated into text messages that are delivered to the users, always according to the goal configuration. As a backup plan in those cases where the extraction of positive insights cannot be achieved, some predefined plain general messages that must always accompany every goal configuration are the pieces of information that will be delivered instead. This implementation will be more specifically detailed in Section 4.1.3.

## 4. Coaching Intervention Tool Details

**Figure 9:**
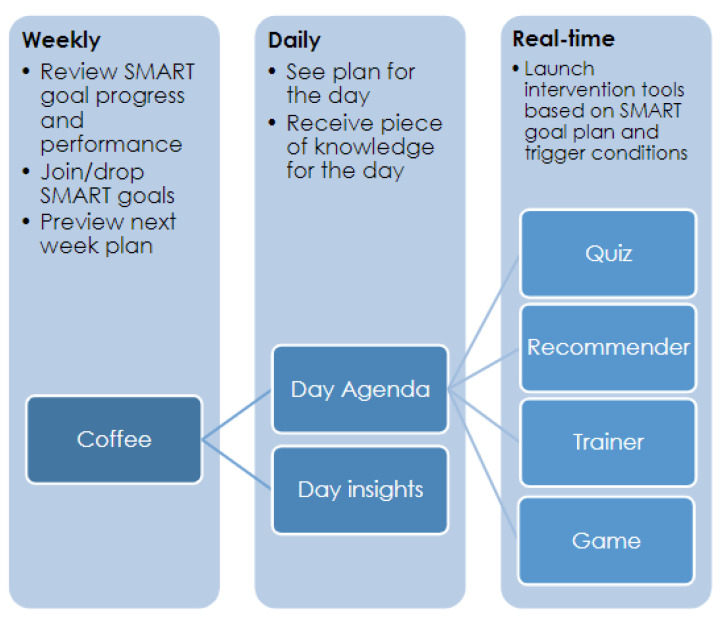
Coaching intervention tools in different timescales.

The intervention tools are the software applications which provide the actual coaching orchestrated by the virtual coach. In this work several example software applications were implemented to provide coaching on the physical, nutritional, cognitive and social areas. On top of them, the *coffee session* application corresponds to the goal management and progress review intervention tool, which is central to the overall coaching process. The *coffee session*, *motivational day agenda* and *day insights* focus more on the awareness and motivation aspects from the I-Change model, while the rest of intervention tools triggered by the VCS are more focused on the specific actions to take towards the behavior change process, even though they may include some motivational and awareness aspects. Figure 9 shows the intervention tool distribution through the coaching timespan.

### 4.1. Core Coaching Intervention Tools

The set of tools described next are core to the system since they organize the coaching process from the user perspective in the different timescales, as previously presented in Figure 7.

#### 4.1.1. Coffee Session

As presented above, the coffee session is an intervention tool to perform reflection about self-performance of the goals during the past week and the goals that the participant has joined. This tool follows a fixed schema with three steps:

1. Review one by one the performance of the active goals with a sequence of charts
2. Give the possibility to join new goals
3. Show a preview of the target activities for the next week

Review performance

This step is carried out per each active goal the participant has joined and it shows two charts, one to depict the overall goal performance and one to show last week’s performance.

**Figure 10:**
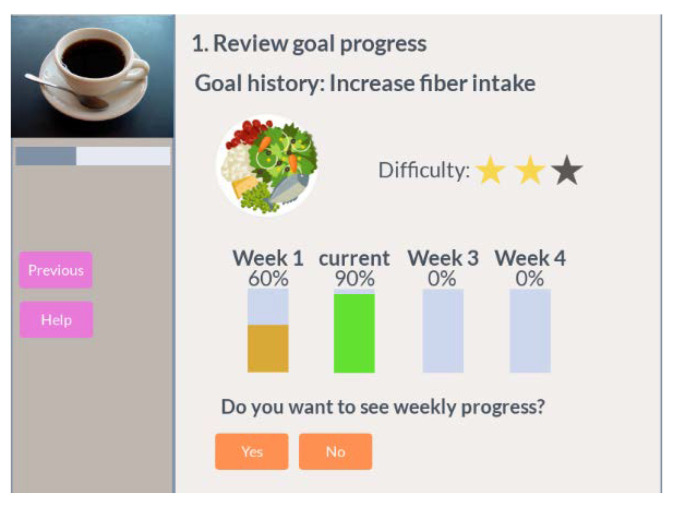
Coffee review progress, overall performance.

An example of an overall goal completion chart, previously introduced in Section 3.4, is shown in Figure 10. There is a bar chart per week. It is filled in based on the number of interventions carried out regarding the maximum target interventions for that week. It is an average for all interventions. The bar color highlights the score of the interventions in that period. Emojis are used to show the score of the interventions (not shown in the figure below).

Then, if the participant wants to see more details, he can review the most recent performance following the same idea for the chart structure where each bar represents one day instead of a whole week. After reviewing the goal, the participant has the option to cancel the goal and move to review the next goal. The participant can skip the review of any goal.

**Figure 11:**
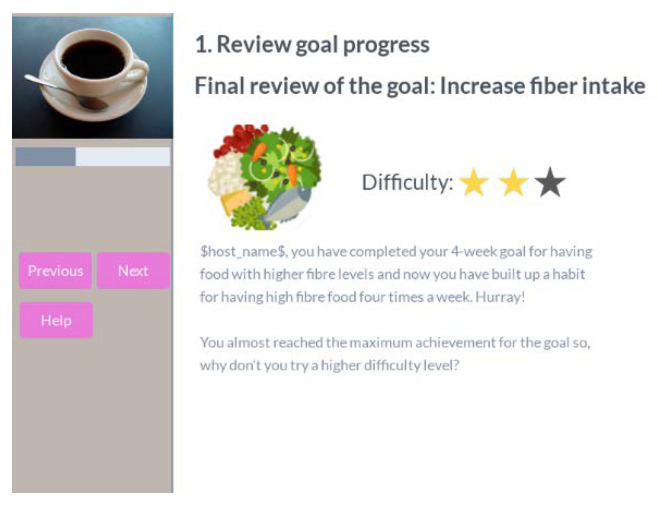
Coffee review progress, goal finish suggestion.

When the participant finishes the whole plan for a goal, he receives a motivational message as well as a suggestion to try a harder or easier difficulty level based on the overall performance, as presented in Figure 11.

**Figure 12:**
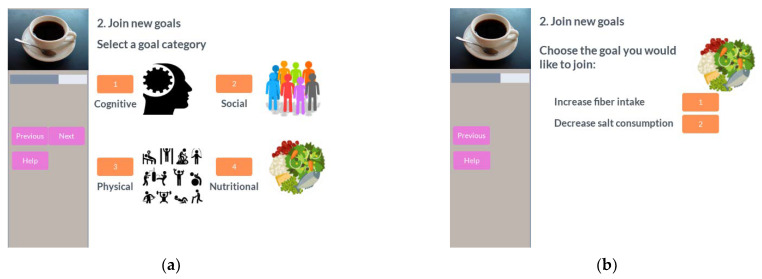
Join goal in coffee session. (**a**) Select goal category; (**b**) select goal in category.

After reviewing the performance of the active goals, the next optional step is to join a new goal. The participant uses a catalogue of predefined plans with different difficulty levels grouped by the coaching dimension they are working on, as shown in Figure 12.

**Figure 13:**
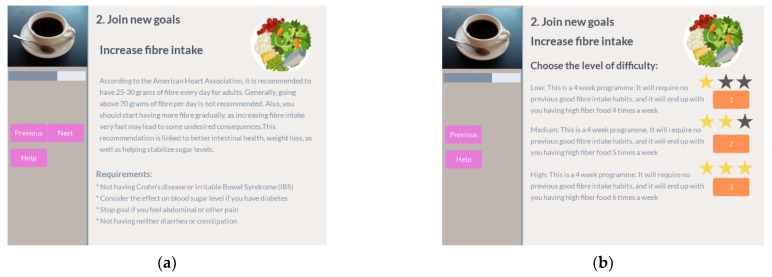
Join goal in coffee session. (**a**) Goal description and requirements; (**b**) set goal difficulty.

Once the participant selects one goal, he can check its description as well as the requirements for the participant to join it. After that, the participant can select the difficulty level for that goal (Figure 13).

**Figure 14:**
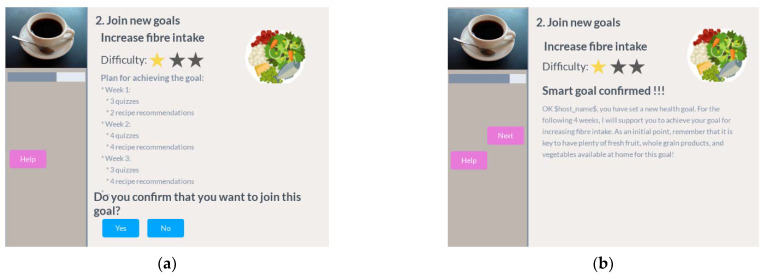
Join goal in coffee session. (**a**) Plan summary and confirmation; (**b**) confirmation and motivational message.

Finally, the user can get a preview of the target activities for that goal per week and confirm that he wants to join the goal. Once confirmed, the participant receives a motivational message (Figure 14).

**Figure 15:**
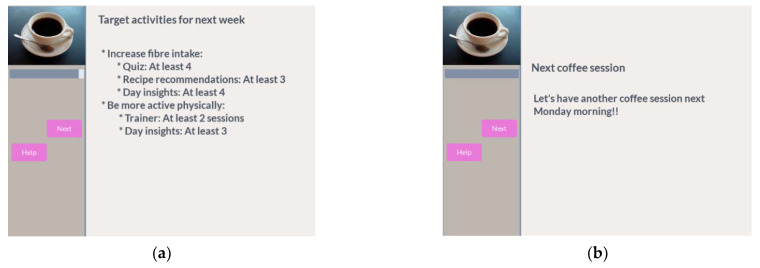
Preview target activities next week and reminder for next week. (**a**) See the target activities for next week; (**b**) reminder for next week’s coffee.

After finishing the goal review and join steps, the participant can have a preview of the overall target activities that will be scheduled the next week for all goals he has joined. He will then receive a reminder to have another coffee session next week (Figure 15).

#### 4.1.2. Motivational Day Agenda

The day agenda is an intervention tool which shows the set of target interventions to be carried out during the day. It does not set a specific time to do them (rationale explained in Section 3.3.3: Momentary planning). This tool is launched the first time the participant is detected every day.

**Figure 16:**
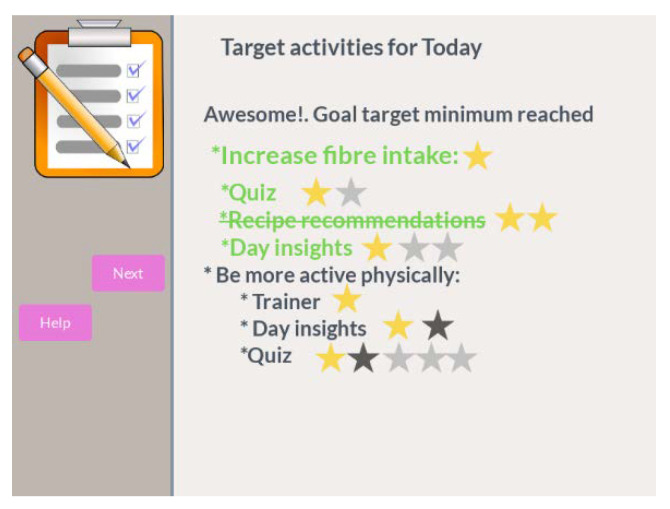
Mock-up for motivational day agenda.

In addition to showing the target interventions for the day, the motivational day agenda is automatically launched whenever the participant achieves a milestone of completing the target interventions for the day with a motivational message, as depicted in Figure 16.

The motivational day agenda can detect the next milestones and produce a motivational message each time any of the following is achieved:

- Minimum interventions for a goal reached
- Maximum interventions for a goal reached
- Minimum interventions for all goals reached
- Maximum intervention for all goals reached

#### 4.1.3. Day Insight

**Figure 17:**
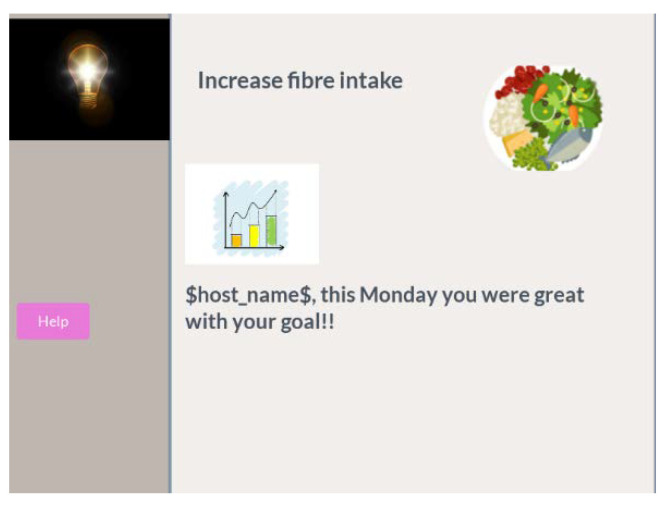
Day insight goal performance.

The day insight is a message presenting a piece of knowledge which is delivered to the participant after the day agenda (Figure 17). There are three kind of day insights based on the data used to obtain them:

- Goal-based insight
- Goal performance analytics day insight
- User model analytics day insight

Goal-based insights are predefined messages contained in the SMART goal plugin to increase the awareness of the participant regarding the objectives of a SMART goal. For example, for the “Increase fiber intake” nutritional SMART goal, one message could be: “Replacing fruit juices with whole fruits is an easy way to increase your fiber intake.”

Goal performance analytics insights compare yesterday’s performance doing the interventions with the participant’s historic performance to produce motivational messages. It also compares the last week against the whole month. For example: “Congratulations!! Yesterday you got the overall best score in your interventions for the increase fiber intake goal.”

User model analytics day insights perform statistical analysis of aggregated participant-sensing data to produce a motivational message. For example: “Lately I see you being more positive. Good for you.” This message would be generated based on the emotion recognition modules. They aggregate data to estimate the participant’s mood and then use statistical tools to identify trends and outliers.

### 4.2. Goal Plan-Dependent Intervention Tools

These action-oriented intervention tools completely depend on the scope of each SMART goal and are not, therefore, key parts of this VCS like the *coffee session*, *motivational day agenda* and *day insights*. They are exchangeable pieces in this system, just like the sensing components and user interface. Nevertheless, several action-oriented intervention tool examples implemented during the CAPTAIN project are presented below.

#### 4.2.1. Gamified Trainer: Physical and Cognitive

The gamified training approach focuses on two pillars: cognitive and physical variants. The physical trainer consists of a large set of gamified physical exercises that are controlled by body movement, recognized by a smart video camera. One can choose from a big pool of gamified exercises and configure the amount per session, as well as the intensity, with different targets ranging from muscle strengthening, stability or endurance. During the gameplay, the participant follows the instructions presented at the beginning of each exercise with text accompanied by a gif image.

Cognitive games show a similar interface, but they target the cognitive coaching dimension.

#### 4.2.2. Recommenders

The system includes several types or recommender systems which basically provide short motivational interventions in the form of messages calling to specific actions offered to the participants in a personalized way. The personalization is based on the participant profile and on the previous interactions with the coach, so it is expected that the suitability of the recommendations and engagement of the participant with the coach increases over time.

The recipe recommender provides the participant with suggestions of healthy recipes aligned with the nutritional SMART goal. The user can see the ingredient list as well as a video and if he does not like the suggestion, he can get additional recipe options.

The social contact recommender is used to deliver motivational messages prompting the participant to contact specific relatives and/or friends to encourage him to communicate with them and improve/maintain his social skills.

The social event recommender aims at encouraging the participants to attend events and activities that are being organized in their surroundings, thus promoting social interaction outside their home.

The physical activity recommender is intended to promote an active lifestyle in the participant through motivational messages encouraging the participant to perform some exercises adapted to the participant’s profile.

The cognitive recommender is designed to deliver motivational messages and recommendations towards fostering the emotional and mental well-being of the participant, thus preserving their mental capacity and quality of life.

#### 4.2.3. Quiz

The quiz is a tool designed to increase the awareness of the user regarding the goal topics. It asks questions to the participant about the goal topics. Besides, it provides an explanation on the rationale behind the correct answer, independent from the answer submitted by the participant.

Contents and difficulty levels are tailored to the active SMART goal(s) and participant profile, respectively, and feedback from the participant about the usefulness of the question proposed is taken into consideration by the coach to further personalize the subsequent quizzes. This tool is based on the spaced learning methodology [27], aiming at maximizing participant’s retention of concepts while minimizing the burden to acquire new knowledge.

## 5. Discussion

As presented in the Introduction, virtual coach systems are still in the early stage of development, far from Siewiorek’s [5] definition. Nevertheless, the H2020 RIA projects funded on virtual coaching for older adults are pushing towards the sophistication of coaching systems. Indeed, an integration of the outcomes of several of these projects combining advanced sensing; natural language interaction; short-, mid- and long-term objective management; and dynamic adaptation and personalization of the coaching process could mostly cover the definition.

Our coach combines SMART goal plans designed and validated by experts on the coaching domain with an adaptive personalization of the intervention delivery provided by the virtual coach. This process employs a combination of data analysis techniques with deterministic algorithms and incremental machine learning algorithms. To our knowledge, this is the first time that this kind of hybrid approach has been presented.

We see several paths to improve our work, from the sensing and user interface aspects to the coach itself and intervention tools. Next, we present and open discussion on the most important improvements.

First, the system coverage is limited currently to older adults’ homes, which constrains sensing capabilities as well as coaching support. It would be desirable to extend this work with at least a companion smartphone application to extend the coverage, and even to the use of wearable devices.

On the sensing aspects, our system currently cannot automatically evaluate the stage in the I-Change behavior change model in which the VCS should put more emphasis. The VCS currently does not have enough sensing capabilities to properly deal with information factors and preceding factors to properly manage the personalization and adaptability of the system to the user preferences. To overcome this limitation, advances on sensing devices and software, as well as the extensive use of data intensive machine learning algorithms, seems to be the path to follow.

On the user interface area, natural-language processing technologies employed by our coach are too basic. It is mandatory to use the latest language processing technologies to keep user engagement. This implies using advanced automatic speech recognition algorithms and speech synthesis technologies. Even more importantly is the use of natural-language understanding for user intent recognition and natural-language generation for conveying information in the most effective and real way. All of them are adapted to the older adult characteristics. Video projection devices also need improvements to deal with the occlusions, strong light sources and mild visual impairment of older adults. Finally, the hardware devices providing these services currently need to be integrated manually in the user’s home and this imposes strong constrains for certain types of houses. Even if they could be embedded into furniture, this process is manual and requires expert technicians for installation and maintenance. In addition, this increases the cost of the overall system and limits its global application. A simplification of the system and evolution of hardware and low-level sensing are required for commercial exploitation.

On the coaching aspects we see that we need a clearer way to map the SMART goal concepts into the coaching plans and everyday coaching. In addition, we should explore alternative approaches to the SMART criteria for goal definition, such as PURE (the right goal, Positively Stated, Understood, Relevant, Ethical) [28]. It is also important to connect and group SMART goals under a long-term goal for the user to have a big picture to foster motivation. We also see that we need to improve the balance between static SMART goal plans and dynamic scheduling by the coach. This is a complex area because making the coach decide the interventions for the user on its own (or modifying pre-defined plans) would make this system a medical device with implications on safety and regulatory aspects. On the other hand, it could permit finer-grain coaching adaptation to the user evolution. Finally, we are not yet taking advantage of the data collected in the server side about population analytics to refine and validate coaching plans nor to improve user experience and personalization.

Regarding the coaching intervention tools, we see a potential market here for software tools and support hardware. Our concept is extendable to cover new tools; nevertheless, for the time being the process of adding new intervention tools is not completely transparent. It requires extending the unified GUI application, designing the interaction flow with it, as well as some modifications of the coach itself.

Finally, a proper long-term validation of this concept implementation is required. Due to COVID-19 restrictions, the extension and coverage of the validation of this implementation was dramatically reduced, therefore it was not included in this paper.

We consider that the preventive virtual coaching concept and ecosystem presented here can be a key piece in the future active and healthy aging systems for independent living, combined with other services such as integration with healthcare, as a telemedicine tool, specifically to manage chronic diseases and pre-frailty stages, monitoring for dangerous situations preventively and in real time, and as an assistive tool for physical and cognitive decline. Such a complete system for ambient assisted living at home would extend healthy years, reduce costs for healthcare and social services and provide better quality of life to older adults.

## 6. Conclusions

This paper presented a virtual coaching concept and the implementation of the concept from the CAPTAIN H2020 (2017–2021) research and innovation action project.

The virtual coach system is grounded on the concepts of SMART goals and the I-Change behavior change mode which aims to empower the older adult in reaching his goals and monitoring his progress, even if he is supported by a caregiver. Coaching covers the cognitive, physical, social and nutritional areas on a preventive well-being level.

The virtual coach is complemented by user sensing devices and hardware, developed during the CAPTAIN project, including speech-based bidirectional interaction with the user and video pico-projectors at home, following edge computing approaches with the support of a server-side control panel.

The design and implementation of the approach permits its extendibility to more use cases and coaching intervention tools, as well as its adaptability to other coaching domains and types of population. It also fosters the creation of a commercial market where third-party institutions can contribute by defining new coaching plans.

Finally, the authors consider that the virtual coaching concept presented here will be part of the future active and healthy aging systems for independent living, combined with other services. It will become a milestone in ambient assisted living at home, which will foster extended healthy years, reduced costs for healthcare and social services and provide better quality of life to older adults.

## Data Availability

Not applicable.
