# Correction: Beristain Iraola et al. User Centered Virtual Coaching for Older Adults at Home Using SMART Goal Plans and I-Change Model. *Int. J. Environ. Res. Public Health* 2021, *18*, 6868

**DOI:** 10.3390/ijerph19042116

**Published:** 2022-02-14

**Authors:** Andoni Beristain Iraola, Roberto Álvarez Sánchez, Santiago Hors-Fraile, Despoina Petsani, Michail Timoleon, Unai Díaz-Orueta, Joanne Carroll, Louise Hopper, Gorka Epelde, Jon Kerexeta, Panagiotis D. Bamidis, Evdokimos I. Konstantinidis

**Affiliations:** 1 Digital Health & Biomedical Technologies Department, Vicomtech Foundation, Basque Research and Technology Alliance (BRTA), Mikeletegi 57, 20009 San Sebastián, Spain; ralvarez@vicomtech.org (R.Á.S.); gepelde@vicomtech.org (G.E.); jkerexeta@vicomtech.org (J.K.); 2 e-Health Department, Biodonostia Health Research Institute, Paseo Dr Begiristain s/n, 20014 San Sebastián, Spain; 3 Salumedia Labs, Research Division of Adhera Health, Inc., Palo Alto, CA 94304, USA; shors@adherahealth.com; 4 Lab of Medical Physics and Digital Innovation, School of Medicine, Aristotle University of Thessaloniki, 54124 Thessaloniki, Greece; despoinapets@gmail.com (D.P.); mtimoleon@auth.gr (M.T.); pdbamidis@gmail.com (P.D.B.); evdokimosk@gmail.com (E.I.K.); 5 Department of Psychology, Maynooth University, Maynooth W23 F2H6, Co. Kildare R51, Ireland; unai.diazorueta@mu.ie; 6 School of Psychology, Dublin City University, Dublin 9, Ireland; joanne.carroll@dcu.ie (J.C.); louise.hopper@dcu.ie (L.H.); 7 WITA SRL, 38123 Trento, Italy

The author would like to change the authorship in the previous publication [1].

## Addition of Authors

**Santiago Hors-Fraile, Despoina Petsani, Michail Timoleon, Unai Díaz-Orueta, Joanne Carroll, Louise Hopper, Gorka Epelde, Jon Kerexeta, Panagiotis D. Bamidis, Evdokimos I. Konstantinidis** were not included as authors in the published article. The corrected Author Contributions Statement appears here. The authors apologize for any inconvenience caused and state that the scientific conclusions are unaffected. The original article has been updated.
